# Immune reconstitution and survival of patients with parvovirus B19 related pure red cell aplasia after haplo-PBSCT

**DOI:** 10.1007/s00277-022-04831-w

**Published:** 2022-04-09

**Authors:** Xiao Zhou, Peiyao Jiang, Lu Gao, Jun Yang, Yu Cai, Yin Tong, Huiying Qiu, Chongmei Huang, Kun Zhou, Xiaowei Xu, Jiahua Niu, Xinxin Xia, Ying Zhang, Chang Shen, Yu Wei, Jie Shao, Xianmin Song, Liping Wan

**Affiliations:** 1 Department of Hematology, Shanghai General Hospital, Shanghai Jiao Tong University School of Medicine, No. 100 Haining Road, Shanghai, 200080 China; 2 Engineering Technology Research Center of Cell Therapy and Clinical Translation, Shanghai Science and Technology Committee (STCSM), No. 100 Haining Road, Shanghai, 200080 China

**Keywords:** Parvovirus B19, Pure red cell aplasia, Immune reconstitution, HLA-haploidentical, Peripheral blood stem cell transplantation

## Abstract

Parvovirus B19 (PvB19) infection and PvB19 related pure red cell aplasia (PRCA) in recipients with allogeneic hematopoietic stem cell transplantation have been reported sporadically. However, clinical studies with large sample sizes are lacking, especially in patients undergoing HLA-haploidentical peripheral blood stem cell transplantation (haplo-PBSCT). In addition, clinical features, immune reconstitution, and outcomes of these patients are not clear. We conducted a retrospective analysis of 164 patients who received haplo-PBSCT with low-dose anti-thymocyte globulin (ATG) plus low-dose posttransplant cyclophosphamide (PTCy)-based regimen as graft-versus-host disease (GVHD) prophylaxis. We analyzed the incidence of PvB19 related PRCA and compared the clinical characteristics, immune reconstitution, incidence of GVHD, relapse rate, and survival between patients with and without PvB19 related PRCA. A total of 14 (8.5%) recipients developed PvB19 related PRCA after a median of 5.3 months after haplo-PBSCT. These patients with PvB19 related PRCA had slower immune reconstitution, but similar incidences of GVHD, relapse rate, and overall survival compared with recipients without PvB19 related PRCA. PvB19 related PRCA indicated relative delayed and poor immune reconstitution of the recipients early after haplo-PBSCT. PvB19 related PRCA had no effects on GVHD, relapse, and survival.

## Introduction

Allogeneic hematopoietic stem cell transplantation (allo-HSCT) from HLA-haploidentical family member has been increasingly performed in recent years. Posttransplant virus infections are common complications, which are associated with increased non-relapse mortality (NRM) after allo-HSCT. Human parvovirus B19 (PvB19), a nonenveloped, single-stranded DNA virus, persists in tonsils, liver, skin, brain, synovial, and testicular tissues as well as bone marrow latently and would reactivate after allo-HSCT [1, 2]. Its seroprevalence among preschool children, young adults, and elderly individuals is estimated to be 15%, 50%, and 85%, respectively [3]. Due to its strong tropism to P antigen of erythroid progenitor cells, the most common clinical manifestations of PvB19 infection in immunocompromised patients are pure red cell aplasia (PRCA), which is characterized as marked reduction or absence of erythroid precursors in bone marrow and severe anemia [4–9].

PvB19 infection and PvB19 related PRCA in recipients of allo-HSCT have been reported sporadically. However, studies on the clinical features, immune reconstitution, and survivals of large-scale patients are lacking, especially in HLA-haploidentical peripheral blood stem cell transplantation (haplo-PBSCT). Therefore, we conducted a retrospective analysis of patients who received haplo-PBSCT, analyzed the incidence of PvB19 related PRCA, and compared the clinical characteristics, immune reconstitution, incidence of graft versus host disease (GVHD), relapse rate, and survival between patients with and without PvB19 related PRCA.

## Patients and methods

### Patients

A total of 164 consecutive patients of haplo-PBSCT enrolled in the study. All patients received peripheral blood stem cells from their HLA haplo-identical family donors from January 2018 through December 2020 in Shanghai Jiao Tong University Affiliated Shanghai General Hospital. Patients’ refined disease risk index and hematopoietic stem cell transplantation comorbidity index (HCT-CI) were scored according to literatures respectively [10, 11].

### Conditioning regimen

Patients with acute myelogenous leukemia (AML) and myelodysplastic syndrome (MDS) received busulfan, fludarabine, and cytarabine-based conditioning regimen. Patients with acute lymphoblastic leukemia (ALL) and non-Hodgkin lymphoma (NHL) received conditioning regimen with total body irradiation (TBI) or busulfan, cyclophosphamide, and etoposide [12].

### GVHD prophylaxis

All patients received rabbit anti-thymocyte globulin (Thymoglobin®, Genzyme Polyclonals S.A.S.) 2.5 mg/kg on day − 2 and day − 1, cyclophosphamide 50 mg/kg on day + 3 (low-dose ATG/PTCy) followed by cyclosporine A 2 mg/kg/d intravenously from day + 4 and mycophenolate mofetil orally 720 mg three times per day from day + 4 to day + 34 for GVHD prophylaxis [12–14].

### Infection prevention and monitoring

Valaciclovir and Posaconazole were given to all patients for prevention of herpes virus and fungus infection [15]. Compound sulfamethoxazole was used for pneumocystis jirovecii pneumonia prophylaxis after hematopoietic recovery posttransplant. Quantitative real-time polymerase chain reaction (PCR) assays for cytomegalovirus (CMV)-DNA and Epstein-bar virus (EBV)-DNA in peripheral blood were performed once or twice per week. The cutoff value was 1 × 10^3^ copies/mL for both viruses.

### Chimerism studies

Quantitative chimerism monitoring was performed by PCR of short-tandem repeats for sorted CD3^+^ T and CD19^+^ B lymphocytes of bone marrow every month after transplant within the first 6 months[16]. The AmpFlSTR Profiler Plus Kit (Applied Biosystems, USA) and ABI PRISM 3130 genetic analyzer were used for amplifications and analyzed, respectively [16].

### Detection of anti PvB19-IgM and PvB19-DNA

According to published criteria and consensus, for patients with PRCA after transplant, their peripheral blood samples were tested for PvB19-DNA and/or anti-PvB19 IgM [9, 17, 18]. PvB19 DNA was detected by using quantitative real-time PCR (Human Parvovirus Real Time PCR Kit, Shanghai Zhi Jiang Biotechnology Company Limited, China). The cutoff value was 1 × 10^3^ copies/mL according to manufacturer. Anti-PvB19 IgM was detected by using Gold Immunofiltration Assay (GIFA, Shandong Kanghua Bio-medical Technology Company Limited, China).

### Statistical analysis

SPSS 25.0 was used for data analysis. Baseline characteristics were summarized using descriptive statistics. Fisher exact and chi-square tests were used to compare categorical variables, and the Wilcoxon rank-sum test was used to compare continuous variables. Lymphocyte counts were analyzed with unpaired *t* test. Overall survival (OS), relapse free survival (RFS), GVHD, and relapse free survival (GRFS) were estimated with Kaplan–Meier and compared by the log-rank test. Risk factors for PvB19 related PRCA were examined in Cox proportional hazards models, and factors with *P* value < 0.200 were included into multivariate analysis. A 2-sided *P* value < 0.050 indicated statistical significance.

## Results

The patients’ characteristics were shown in Table 1. There were 105 male and 59 female patients. Their median age was 39 (6–64) years old. There were 87 (53.0%) patients diagnosed with AML, 37 (25.0%) patients with ALL, 23 (14.0%) patients with MDS, and 17 (10.4%) patients with NHL.

**Table 1:** Patient characteristics

Variables	All patients (*N* = 164)	Patients with PvB19 related PRCA (*N* = 14)	Patients without PvB19 related PRCA (*N* = 150)	*P* value
Recipient age, yr, *n* (%)				0.085
< 39	81 (49.4)	10 (6.1)	71 (43.3)	
≥ 39	83 (50.6)	4 (2.4)	79(48.2)	
Recipient sex, *n* (%)				0.546
Male	105 (64.1)	10 (6.1)	95 (58.0)	
Female	59 (35.9)	4 (2.4)	55 (33.5)	
Diagnosis, *n* (%)				< 0.001
AML	87 (53.0)	3 (1.8)	84 (51.2)	
B-ALL	30 (18.3)	9 (5.5)	21 (12.8)	
T-ALL	7 (4.3)	0	7 (4.3)	
MDS	23 (14.0)	2 (1.2)	21 (12.8)	
NHL	17 (10.4)	0	17 (10.4)	
R-DRI, *n* (%)				0.158
Low	13 (7.9)	3 (1.8)	10 (6.1)	
Intermediate	126 (76.9)	8 (4.9)	118 (72.0)	
High	25 (15.2)	3 (1.8)	22 (13.4)	
Very high	0	0	0	
Disease status, *n* (%)				0.540
CR	111 (67.7)	11 (6.7)	100 (61.0)	
NR	53 (32.3)	3 (1.8)	50 (30.5)	
HCT-CI, *n* (%)				0.899
< 3	148 (90.2)	12 (7.3)	136 (82.9)	
≥ 3	16 (9.8)	2 (1.2)	14 (8.6)	
Donor age, yr, *n* (%)				1.000
< 40	116 (70.7)	10 (6.1)	106 (64.6)	
≥ 40	48 (29.3)	4 (2.5)	44 (26.8)	
Donor-recipient sex match, *n* (%)				0.773
Female to male	36 (21.9)	4 (2.4)	32 (19.5)	
Others	128 (78.1)	10 (6.1)	118 (72.0)	
Donor, *n* (%)				0.822
Parents	45 (27.4)	4 (2.4)	41 (25.0)	
Sibling	33 (20.1)	3 (1.8)	30 (18.3)	
Offspring	81 (49.4)	7 (4.3)	74 (45.1)	
Cousin	5 (3.1)	0	5 (3.1)	
ABO blood type, *n* (%)				0.221
Compatible	83 (50.6)	6 (3.7)	77 (46.9)	
Minor incompatible	39 (23.8)	6 (3.7)	33 (20.1)	
Major or bidirectional incompatible	42 (25.6)	2 (1.2)	40 (24.4)	
Conditioning regimen, *n* (%)				0.580
MAC	140 (85.4)	13(7.9)	127(77.5)	
RIC	24 (14.6)	1(0.6)	23(14.0)	
CD34 + cells in graft, *n* (%)				0.831
< 8 × 10^6^/kg	45 (27.4)	3 (1.8)	42 (25.6)	
≥ 8 × 10^6^/kg	119 (72.6)	11 (6.7)	108 (65.9)	
Umbilical cord blood, *n* (%)				0.962
Yes	83 (50.6)	7 (4.3)	76 (46.3)	
No	81 (49.4)	7 (4.3)	74 (45.1)	

*PvB19* parvovirus B19, *PRCA* pure red cell aplasia, *AML* acute myeloid leukemia, *B-ALL* B-cell acute lymphoblastic leukemia, *T-ALL* T-cell acute lymphoblastic leukemia, *MDS* myelodysplastic syndrome, *NHL* non-Hodgkin’s lymphoma, *R-DRI* refined-disease risk index, *CR* complete remission, *NR* non-remission, *HCT-CI* hematopoietic cell transplantation comorbidity index, *PBSC* peripheric blood stem cell, *MAC* myeloablative conditioning, *RIC* reduced-intensity conditioning

### Engraftment

Out of the 164 patients, 158 (96.3%) patients had successful engraftment; 6 patients died within 28 days. Four of them died of severe pulmonary infection; another 2 patients died of grade IV aGVHD and heart failure, respectively. No patients had primary graft failure. The median time to the engraftment of neutrophil and platelet was 13 (10 ~ 21) and 15 (12 ~ 26) days, respectively. Of the 158 patients, 150 (94.9%) recipients achieved full donor chimerism within 28 days after transplantation.

### PvB19 related PRCA

A total of 14 (8.5%) recipients were diagnosed with PvB19 related PRCA after a median of 5.3 (1.1 ~ 34.5) months posttransplant based on their clinical manifestations and serum PvB19-DNA viral loads. One patient had systemic PvB19 infection including PRCA and pneumonia. There were 6 recipients with viral loads ≤ 1 × 10^8^ copies/mL and 8 recipients > 1 × 10^8^ copies/mL. Only 4 patients’ peripheral blood samples were tested for anti-PvB19 IgM, including 1 positive and 3 negatives. ABO blood group incompatible related PRCA were excluded from these patients. All of the patients achieved complete donor chimerism. The median hemoglobin and reticulocyte count were 45 (25 ~ 69) g/L and 0.17 (0.09 ~ 0.38) × 10^9^/L. All patients received high dose intravenous immunoglobulin (IVIG, 400 mg/kg/d × 5 d) therapy. Twelve (85.7%) patients had obvious improvement of hemoglobin level (≥ 30 g/L increasement than pre-treatment) in 1 month after treatment, including 6 patients with viral load ≤ 1 × 10^8^ copies/mL and 6 patients > 1 × 10^8^ copies/mL. One patient with viral load > 1 × 10^8^ copies/mL had no response until 3 months after IVIG treatment. Only the patient with systemic PvB19 infection had no response and died 42 days after IVIG treatment. After 1-year follow-up, 11 patients had complete remission of PRCA; however, another 2 (14.3%) patients with PvB19 DNA above 1 × 10^8^ copies/mL had recurrence of PvB19 related PRCA 3.5 months and 4.3 months after the first episode, respectively, but they also had complete remission of PRCA again within 2 months after repeated IVIG.

### Risk factors of PvB19 related PRCA

We compared clinical characteristics between patients with and without PvB19 related PRCA (Table 1). The primary diagnosis of patients with and without PvB19 related PRCA was different (*P* < 0.001). The proportion of B-cell acute lymphoblastic leukemia (B-ALL) in patients with PvB19 related PRCA was significantly higher than patients without PvB19 related PRCA (64.3% vs. 14.0%, *P* < 0.001).

Thirteen clinical factors were taken into univariate analysis for PvB19 related PRCA. Factors with *P* value < 0.020 were included in multivariable analysis. Among them, lymphoid hematological malignancies (RR = 4.292, *P* = 0.006) and HCT-CI ≥ 3 (RR = 9.010, *P* = 0.007) were independent risk factors for PvB19 related PRCA (Table 2).

**Table 2:** Univariate and multivariate analysis for patients with PvB19 related PRCA

Variable	Univariate analysis			Multivariate analysis		
	HR	95%CI	*P* value	RR	95%CI	*P* value
Recipient age (< 39 vs. ≥ 39, yr)	2.236	0.784 ~ 6.378	0.232			
Recipient sex (male vs. female)	1.384	0.469 ~ 4.120	0.560			
Diagnosis (lymphoid vs. myeloid)	4.600	1.488 ~ 14.220	0.008	4.292	1.566 ~ 15.519	0.006
R-DRI (L + I vs. H + V)	0.619	0.144 ~ 2.659	0.519			
Disease status (CR vs. NR)	1.669	0.547 ~ 5.098	0.369			
HCT-CI (≥ 3 vs. < 3, scores)	40.510	1.985 ~ 826.500	0.016	9.010	1.844 ~ 44.023	0.007
Donor age (< 40 vs. ≥ 40, yr)	1.058	0.337 ~ 3.328	0.923			
Donor-recipient sex match (others vs. female to male)	0.677	0.191 ~ 2.402	0.550			
Donor sources (parents, offspring and sibling vs. cousin)	2.809	0.142 ~ 55.690	0.498			
ABO blood type (compatible vs. incompatible)	0.725	0.254 ~ 2.068	0.548			
Conditioning regimen (MAC vs. RIC)	1.846	0.424 ~ 8.028	0.414			
PBSC CD34 + cells (< 8 vs. ≥ 8, 10^6^/kg)	0.719	0.224 ~ 2.308	0.580			
Umbilical cord blood (no vs. yes)	1.049	0.368 ~ 2.993	0.928			

*PvB19* parvovirus B19, *PRCA* pure red cell aplasia, *Myeloid* acute myeloid leukemia and myelodysplastic syndrome, *Lymphoid* acute lymphoblastic leukemia and non-nodgkin’s lymphoma, *R-DRI*: refined disease risk index, *L* low, *I* intermediate, *H* high, *V* very high, *CR* complete remission, *NR* non-remission, *HCT-CI* hematopoietic cell transplantation comorbidity index, *PBSC* peripheral blood stem cell, *MAC* myeloablative conditioning, *RIC* reduced-intensity conditioning

### Immune reconstitution

**Fig. 1:**
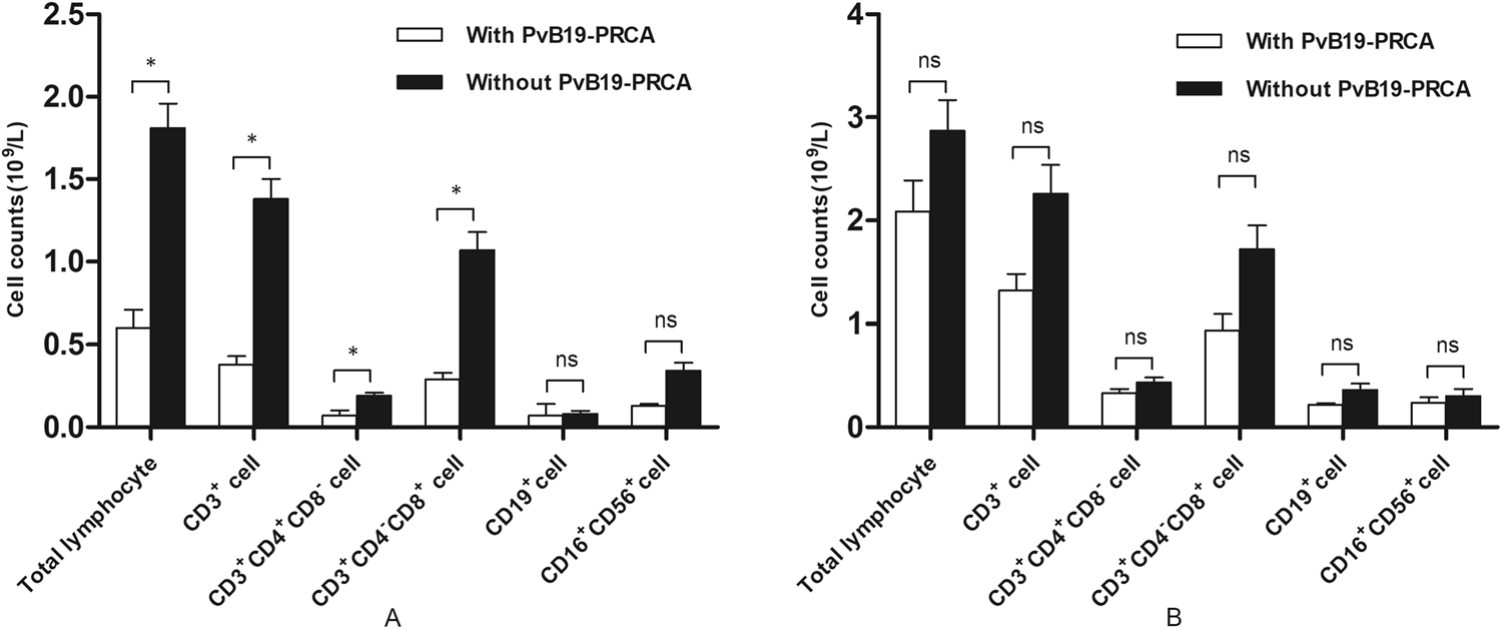
Comparison of lymphocytes subset counts on the 3rd and 12th months after transplantation. **A**: Lymphocytes subset counts on the 3rd month; **B**: Lymphocytes subset counts on the 12th month

**Fig. 2:**
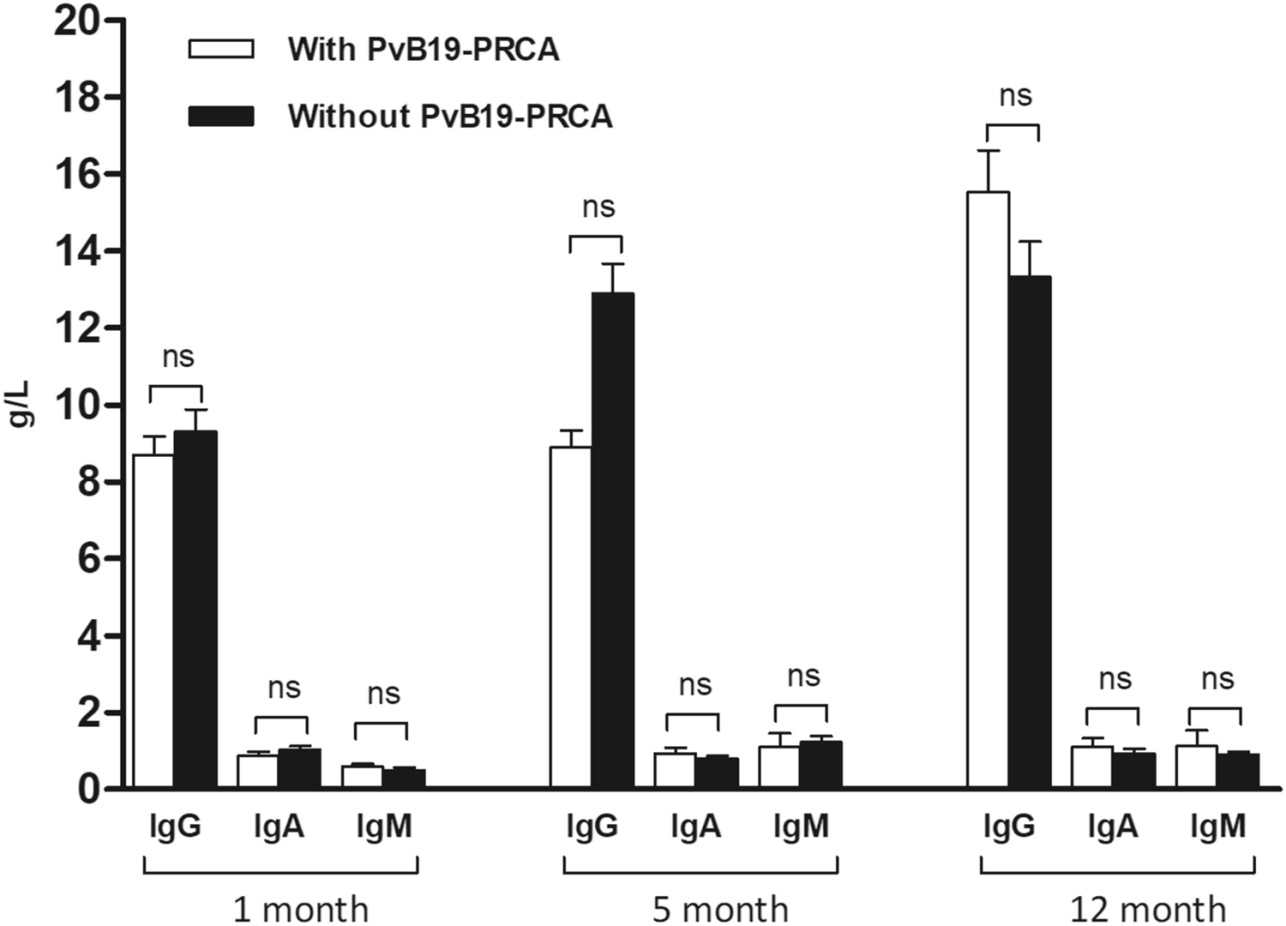
Levels of IgG, IgM, and IgA on the 1st, 5th, and 12th months after transplantation

We compared lymphocyte subset counts of patients with and without PvB19 related PRCA on the 3rd, 5th, and 12th months after haplo-PBSCT. On the 3rd month and 5th month posttransplant, patients with PvB19 related PRCA had significantly lower total lymphocytes (*P* = 0.018 and 0.002), CD3 + (*P* = 0.022 and 0.003), CD4^+^ (*P* = 0.045 and 0.020), and CD8^+^ (*P* = 0.033 and 0.006) lymphocyte counts (Fig. 1A and Table 3). Moreover, on the 5th month posttransplant, patients with PvB19 related PRCA also had less CD19^+^ (*P* = 0.059), CD4 + CD25^+^ (*P* < 0.001), CD4^+^CD45RO^+^ (*P* = 0.044), and CD8^+^CD45RO^+^ (*P* = 0.035) lymphocyte counts (Table 3). However, on the 12th month posttransplant, the total and lymphocyte subset counts in patients with and without PvB19 related PRCA were similar (Fig. 1B). Other than that, serum levels of IgG, IgM, and IgA in patients with PvB19 related PRCA were relatively lower than those in patients without PvB19 related PRCA on the 5th month after transplantation, but no significant difference was found. Additional, the serum levels of IgG, IgM, IgA were similar between patients with and without PvB19 related PRCA on the 1st month and 12th month posttransplant (Fig. 2).

**Table 3:** Comparison of lymphocytes subset counts of patients with and without PvB19 related PRCA on the 5th months posttransplant

Lymphocyte subsets (M ± SEM, 95%CI)	Patients with PvB19 related PRCA (*N* = 10)	Patients without PvB19 related PRCA (*N* = 54)	*t* value	*P* value
Total lymphocyte (10^9^/L)	1.03 ± 0.18 (0.65 ~ 1.42)	2.22 ± 0.18 (1.86 ~ 2.57)	3.30	0.002
CD3^+^ (10^9^/L)	0.72 ± 0.14 (0.41 ~ 1.0)	1.69 ± 0.16 (1.37 ~ 2.00)	3.05	0.003
CD3^+^CD4^+^CD8^−^ (10^9^/L)	0.14 ± 0.05 (0.04 ~ 0.24)	0.24 ± 0.02 (0.20 ~ 0.27)	2.38	0.020
CD3^+^CD4^−^CD8^+^ (10^9^/L)	0.53 ± 0.10 (0.31 ~ 0.74)	1.33 ± 0.14 (1.05 ~ 1.61)	2.85	0.006
CD4/CD8	0.28 ± 0.05 (0.16 ~ 0.39)	0.26 ± 0.03 (0.20 ~ 0.32)	0.33	0.743
CD4^+^CD25^+^ (10^6^/L)	2.09 ± 0.78 (0.41 ~ 3.77)	13.30 ± 1.37 (10.54 ~ 16.05)	4.09	< 0.001
CD8^+^CD25^+^ (10^6^/L)	1.38 ± 0.64 (− 0.01 ~ 2.76)	1.77 ± 0.20 (1.36 ~ 2.18)	0.77	0.445
CD3^+^CD69^+^ (10^6^/L)	32.14 ± 10.78 (8.86 ~ 55.42)	64.98 ± 25.00 (14.85 ~ 115.10)	0.66	0.511
CD3^+^HLA-DR^+^ (10^6^/L)	87.58 ± 18.88 (46.78 ~ 128.40)	198.40 ± 21.76 (154.80 ~ 242.10)	2.52	0.014
CD4^+^CD45RA^+^ (10^6^/L)	2.65 ± 0.75 (1.03 ~ 4.27)	10.64 ± 1.56 (7.51 ~ 13.76)	2.58	0.012
CD4^+^CD45RO^+^ (10^6^/L)	134.50 ± 46.08 (34.98 ~ 234.10)	217.20 ± 16.78 (183.60 ~ 1250.90)	2.05	0.044
CD8^+^CD45RA^+^ (10^6^/L)	101.00 ± 47.50 (− 1.67 ~ 203.60)	342.90 ± 43.11(256.40 ~ 429.40)	2.74	0.008
CD8^+^CD45RO^+^ (10^6^/L)	411.40 ± 83.99 (229.90 ~ 592.90)	888.70 ± 110.30 (667.40 ~ 1110.00)	2.15	0.035
CD4^+^CD29^+^ (10^6^/L)	109.60 ± 34.80 (34.40 ~ 184.80)	194.80 ± 18.79 (157.10 ~ 232.50)	2.08	0.041
CD8^+^CD28^+^ (10^6^/L)	118.50 ± 34.81(43.26 ~ 193.60)	215.00 ± 17.97 (179.00 ~ 251.10)	2.45	0.017
CD19^+^ (10^9^/L)	0.06 ± 0.02 (0.02 ~ 0.09)	0.12 ± 0.02 (0.09 ~ 0.16)	1.92	0.059
CD16^+^CD56^+^ (10^9^/L)	0.24 ± 0.07 (0.09 ~ 0.38)	0.38 ± 0.05 (0.28 ~ 0.48)	1.41	0.164

*PvB19* parvovirus B19, *PRCA* pure red cell aplasia, *M* mean, *SEM* standard error of mean

### GVHD

A total of 25 (15.2%) patients developed grade II–IV aGVHD within 100 days. The median onset time of aGVHD was 18 (9 ~ 95) days posttransplant. In patients with PvB19 related PRCA, 2 (14.3%) patients developed grade II–IV aGVHD within 100 days (Table 4). Both of the patients had intensified GVHD therapy when they were diagnosed with PvB19 related PRCA. In terms of the incidences of grade II–IV and III–IV aGVHD, 1-year cGVHD and moderate to severe cGVHD, there were no significant differences between patients with and without PvB19 related PRCA (14.3% vs.15.3%, *P* = 0.916; 7.1% vs. 4.0%, *P* = 0.607; 14.3% vs. 21.2%, *P* = 0.781 and 7.1% vs. 15.3%, *P* = 0.664; respectively) (Table 4).

**Table 4:** Comparison of incidences of GVHD in patients with and without PvB19 related PRCA

GVHD, *n* (%)	All patients (*n* = 164)	Patients with PvB19 related PRCA	Patients without PvB19 related PRCA	*P* value
		(*n* = 14)	(*n* = 150)	
II–IV aGVHD	25 (15.2)	2 (14.3)	23 (15.3)	0.916
III–IV aGVHD	8 (4.3)	1 (7.1)	6 (4.0)	0.607
1 year-cGVHD	34 (20.6)	2 (14.3)	32 (21.2)	0.781
1 year-moderate to severe cGVHD	24 (14.6)	1 (7.1)	23 (15.3)	0.664

*GVHD* graft-versus-host disease, *PvB19* parvovirus B19, *PRCA* pure red cell aplasia

### CMV, EBV, and BKV infection

The incidences of CMV, EBV, and BK virus (BKV) infection were similar between patients with and without PvB19 related PRCA within 5 months posttransplant (42.9% vs. 39.3%, *P* = 0.797; 28.6% vs. 28.7%, *P* = 0.994; 21.4% vs. 12.7%, *P* = 0.610) (Table 5).

**Table 5:** Comparison of incidences of CMV, EBV, and BKV infection in patients with and without PvB19 related PRCA within 5 months posttransplant

Viremia, *n* (%)	All patients (*n* = 164)	Patients with PvB19 related PRCA	Patients without PvB19 related PRCA	*P* value
		(*n* = 14)	(*n* = 150)	
CMV	65 (39.6)	6 (42.9)	59 (39.3)	0.797
EBV	47 (28.7)	4 (28.6)	43 (28.7)	0.994
BKV	22 (13.4)	3 (21.4)	19 (12.7)	0.610

*PvB19* parvovirus B19, *PRCA* pure red cell aplasia, *CMV* cytomegalovirus, *EBV* Epstein-bar virus, *BKV* BK virus

### Relapse

**Fig. 3:**
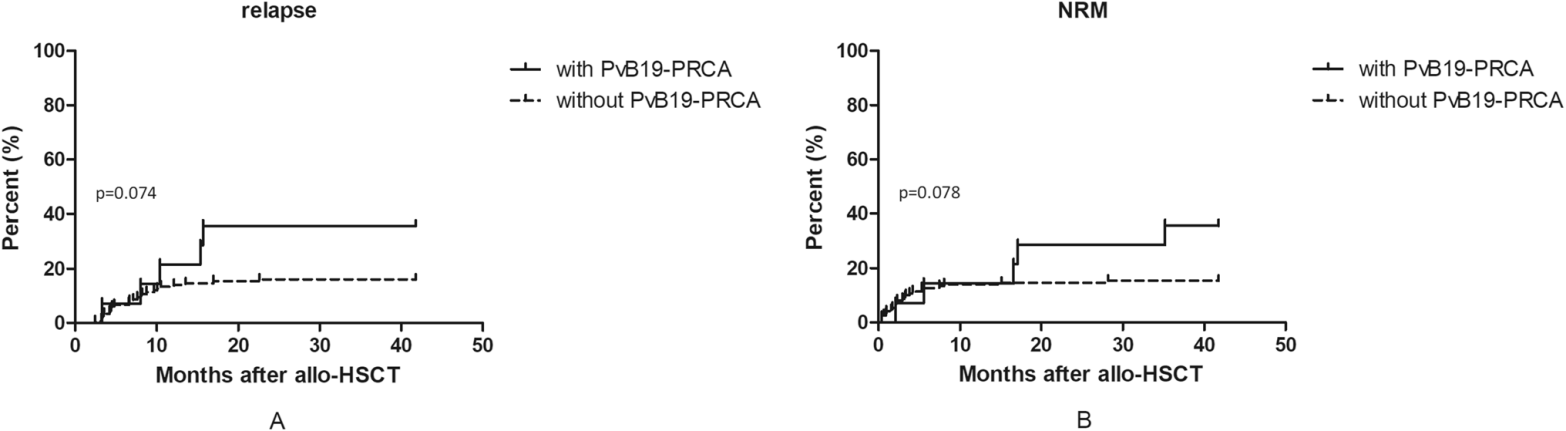
Cumulative incidences of relapse and non-relapse mortality (NRM) of patients with and without parvovirus B19 related pure red cell aplasia. **A**: Cumulative incidence of relapse; **B**: Cumulative incidence of NRM

After a median of 13.9 (0.4 ~ 41.8) months follow-up, 29 patients relapsed. The median time to relapse was 7.6 (2.5 ~ 22.6) months. In patients with PvB19 related PRCA, 5/14 (35.7%) patients relapsed. Three of them had PvB19 related PRCA, and diseases relapse simultaneously. The other two patients had disease relapse within 2 months after the diagnosis of PvB19 related PRCA. Compared with patients without PvB19 related PRCA, the 1-year relapse rate in patients with PvB19 related PRCA was relatively higher; however, there were no significant differences (21.4% vs. 13.3%, *P* = 0.074) (Fig. 3A).

### Survival

**Fig. 4:**
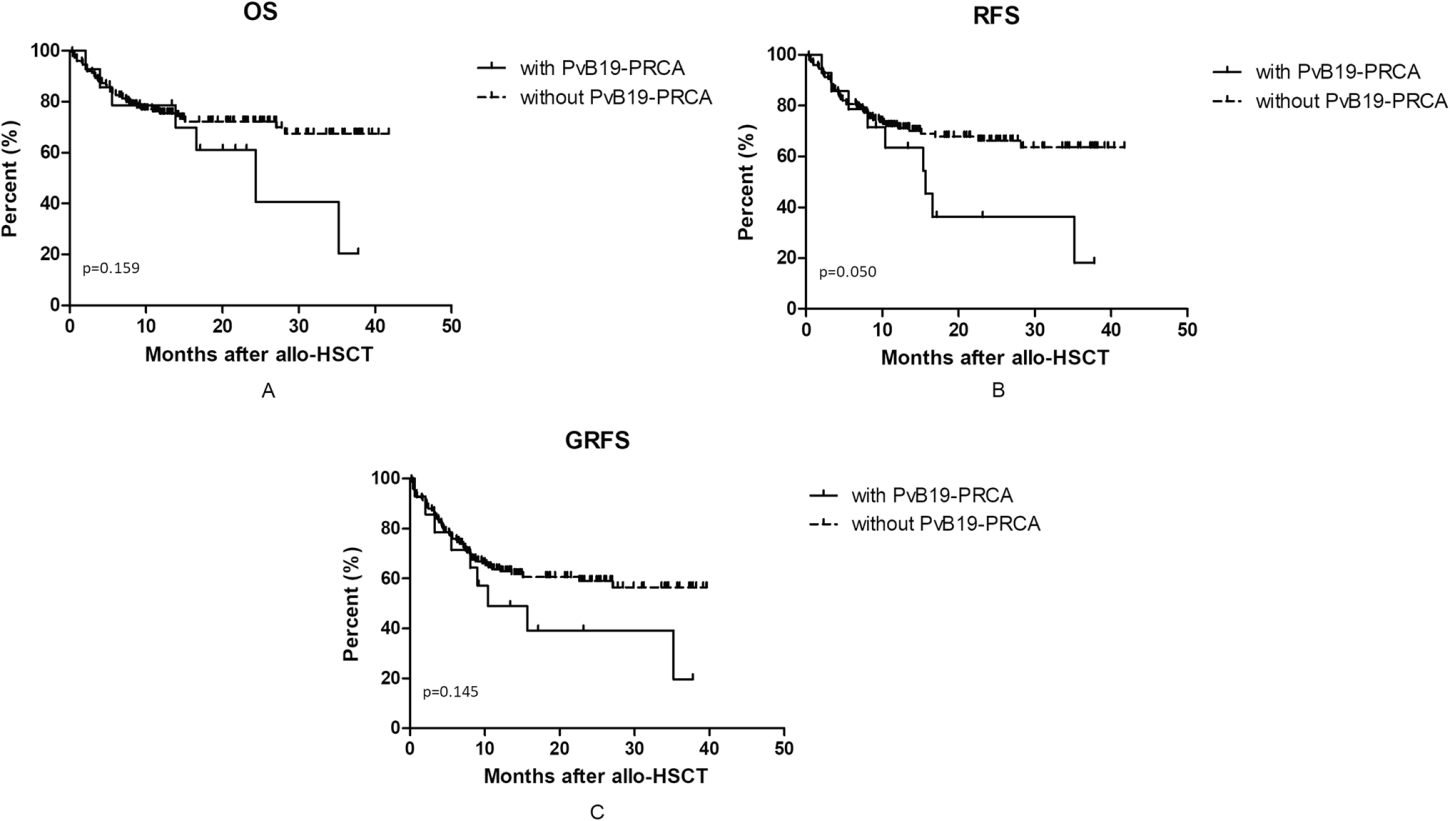
Survival of patients with and without parvovirus B19 related pure red cell aplasia. **A**: Overall survival (OS); **B**: Relapse-free survival (RFS); C: GvHD-free, relapse-free survival (GRFS)

By the end of follow-up, 49 patients died; 39 (79.6%) of them died within 1 year. In patients with PvB19 related PRCA, 8/14 (57.1%) patients died, 5 of them died of infection (including one patient died of disseminated PvB19 infection), and 3 of them died of disease relapse. In patients without PvB19 related PRCA, 41/150 (27.3%) patients died. Eighteen of them died of disease relapse, 16 died of infection, 2 died of cerebral hemorrhage, 2 died of graft rejection, 1 died of heart failure, 1 died of grade IV aGVHD, and 1 of severe cGVHD. The 1-year NRM, OS, RFS, and GRFS were similar between patients with and without PRCA (14.2% vs. 14.0%, *P* = 0.078; 78.6% vs. 75.4%, *P* = 0.159; 63.5% vs. 71.2%, *P* = 0.050; 48.9% vs. 62.8%, *P* = 0.145; respectively) (Figs. 3B, 4A, B, and C).

## Discussion

In this study, we analyzed data of 164 recipients of haplo-PBSCT with low-dose ATG/PTCy followed by CSA/MMF for GVHD prophylaxis. Fourteen (8.5%) recipients developed PvB19 related PRCA posttransplant after a median of 5.3 months. Patients with lymphoid hematological malignancies especially B-ALL or HCT-CI ≥ 3 had higher risk for PvB19 related PRCA. Compared with patients without PvB19 related PRCA, patients with PvB19 related PRCA had slower immune reconstitution, similar incidences of acute and chronic GVHD, relapse rate, OS, RFS, and GRFS.

There were some case reports of PvB19 related PRCA after allo-HSCT sporadically; however, the incidence and clinical outcomes in recipients of allo-HSCT especially haplo-PBSCT were lacking. Nevertheless, there were many studies on the incidence of PvB19 related PRCA in solid organ transplantation. It was reported that the incidences of PvB19 related PRCA in adult and pediatric liver transplant recipients were about 2.3% [19, 20] and 9.3% respectively [21]. In renal transplant recipients, a meta-analysis showed the prevalence was 7.6% [22]. The incidence was similar between our study and reported data in liver and renal transplantation.

We found patients with lymphoid hematological malignancies especially B-ALL or HCT-CI ≥ 3 had higher risk for PvB19 related PRCA. The reason might be the lymphodepleting regimen for B-ALL with cyclophosphamide and fludarabine in our study, which resulted in severe immune deficiency after transplant. Previous study found higher HCT-CI score was closely related to increased risk of viral infection [23]; our study was in accordance with it. Since there was no specific antiviral agent for PvB19, present treatments for PvB19 related PRCA included tapering of immunosuppression and high dose IVIG [24, 25]. In a retrospective study on 10 immunocompromised patients, Crabol et al. reported 90% patients had improvement of hemoglobin levels after one course of IVIG treatment. However, about 30% patients had recurrence of PvB19 related PRCA within a median of 4 months [25]. Our study showed similar response rate and relatively lower recurrent rate based on larger case data.

In our study, patients with PvB19 related PRCA had significantly lower counts of CD3^+^, CD4^+^, CD8^+^, CD4^+^CD45RO^+^, and CD8^+^CD45RO^+^ T lymphocyte and CD19^+^ B lymphocyte on the 5th month posttransplant. Mccuedy et al. reported that the median counts of CD4^+^, CD8^+^ T lymphocytes, and CD19^+^ B lymphocytes were 0.2 × 10^9^/L, 0.4 × 10^9^/L, and 0.1 × 10^9^/L on the 6th month after haplo-PBSCT [26]. In the present study, the median counts of lymphocyte subsets in patients without PvB19 related PRCA were consistent with the report above, whereas patients with PvB19 related PRCA had significantly less lymphocytes subset posttransplant. It was reported that recipients after solid organ transplantation or allo-HSCT with significantly lower counts of CD3^+^, CD4^+^, and CD8^+^ T cell were prone to PvB19 infection, and about 99% of immunocompromised patients with PvB19 infection would develop PRCA due to reduction of antiviral T cells and lack of diversity of T cell receptor [6, 27]. Vassiliki et al. also found virus infection was negatively associated with the number of CD4^+^ (*P* = 0.030), CD8^+^ cells (*P* = 0.030), CD4^+^CD45RO^+^ cells (*P* = 0.030), and CD8^+^CD45RO^+^cells (*P* = 0.050) after umbilical cord blood transplantation [28]. In addition, previous study showed insufficient quantity and quality of B cell after transplantation were also responsible for the persistence and recurrence of PvB19 infection [29]. These patients could not produce sufficient immunoglobulin, which leads to chronic and long-term infection of PvB19 [30].

The incidences of grade II–IV aGVHD and 1-year moderate to severe cGVHD were similar in patients with and without PvB19 related PRCA in our study. We supposed it was due to the effective GVHD prophylaxis regimen with low-dose ATG/PTCy in our study [31]. Besides, patients with PvB19 related PRCA had similar total lymphocytes and subsets counts on the 12th month after transplantation. This might be the major reason for similar incidence of cGVHD in patients with and without PvB19 related PRCA.

Relapse rate and overall survival in patients with and without PvB19 related PRCA were similar in this study. Reports about direct influences of PvB19 related PRCA on relapse and survival after allo-HSCT are very rare. In renal transplantation, PvB19 related PRCA has no significant effect on long-term posttransplant survival [32]. PvB19 related PRCA was rarely life-threatening in the majority of cases except for disseminated PvB19 infection [9]. It is a complication closely related to immunocompromised status early posttransplant. In other words, infection with PvB19 indicates relatively delayed and poor immune reconstitution of the recipients early after transplantation, but immunity would gradually restore in the long run. The lymphocytes were lower in the early stage but gradually reconstituted in the later stage in patients with PvB19 related PRCA in our study.

In conclusion, our study showed that 8.5% recipients of haplo-PBSCT developed PvB19 related PRCA after a median of 5.3 months posttransplant, which indicated lower immune status of the recipients early after transplantation, but PvB19 related PRCA had no direct influences on GVHD, relapse, and survival.
